# Supplementary material for: The Effects of Cellular Membrane Damage on the Long-Term Storage and Adhesion of Probiotic Bacteria in Caco-2 Cell Line
Source: Nutrients. 2023 Aug 7;15(15):3484. doi: 10.3390/nu15153484 (PMC10421378; doi:10.3390/nu15153484)
Supplement: Supplementary file 1 [file nutrients-15-03484-s001.zip › nutrients-2530568-supplementary.pdf]

Table S1. Dried samples

sample	m0	m1	m2	m3	m6	m9	m12
E. Faecium - 20 pow	8.73E+09	8.49E+09	9.95E+09	8.12E+09	6.18E+08	7.85E+07	<10^6
EF -20 N2	8.73E+09	9.41E+09	8.98E+09	8.20E+09	2.90E+09	4.61E+07	6.64E+06
EF -20 V	8.73E+09	7.09E+09	8.74E+09	7.58E+09	9.82E+08	2.80E+07	5.45E+06
EF 4 pow	8.73E+09	8.59E+09	8.76E+08	2.98E+08	8.98E+06	<10^6	<10^6
EF 4 N2	8.73E+09	9.39E+09	9.28E+08	3.86E+08	6.03E+07	4.02E+06	<10^6
EF 4 V	8.73E+09	8.83E+09	8.28E+08	3.04E+08	7.00E+07	3.81E+06	<10^6
EF 20 pow	8.73E+09	6.00E+09	7.99E+08	8.32E+07	<10^6	<10^6	<10^6
EF 20 N2	8.73E+09	6.38E+09	6.89E+08	1.92E+08	<10^6	<10^6	<10^6
EF 20 V	8.73E+09	5.43E+09	7.12E+08	9.50E+07	<10^6	<10^6	<10^6
LM -20 pow	5.55E+09	5.36E+09	4.68E+09	3.66E+09	1.85E+08	3.06E+07	<10^6
LM -20 N2	5.55E+09	4.92E+09	5.32E+09	4.80E+09	7.36E+08	1.20E+08	4.95E+06
LM -20 V	5.55E+09	4.65E+09	4.99E+09	4.22E+09	8.19E+08	8.08E+07	2.88E+06
LM 4 pow	5.55E+09	5.55E+09	8.77E+08	2.90E+08	2.75E+07	<10^6	<10^6
LM 4 N2	5.55E+09	4.96E+09	7.90E+08	3.92E+08	4.84E+07	<10^6	<10^6
LM 4 V	5.55E+09	5.64E+09	7.68E+08	4.06E+08	3.82E+07	<10^6	<10^6
LM 20 pow	5.55E+09	2.86E+09	4.98E+08	6.30E+07	<10^6	<10^6	<10^6
LM 20 N2	5.55E+09	3.39E+09	5.10E+08	8.42E+07	<10^6	<10^6	<10^6
LM 20 V	5.55E+09	3.16E+09	5.70E+08	8.10E+07	<10^6	<10^6	<10^6

Table S2. Coated samples

sample	m0	m1	m2	m3	m6	m9	m12
EF -20 pow	9.42E+08	8.54E+08	7.30E+08	5.27E+08	8.73E+07	6.28E+06	<10^6
EF -20 N2	9.42E+08	9.93E+08	9.78E+08	7.28E+08	2.09E+08	9.35E+07	2.90E+07
EF -20 V	9.42E+08	8.57E+08	8.39E+08	8.37E+08	2.89E+08	8.92E+07	9.21E+06
EF 4 pow	9.42E+08	9.20E+08	7.82E+08	3.68E+08	1.85E+07	<10^6	<10^6
EF 4 N2	9.42E+08	7.83E+08	6.73E+08	4.89E+08	7.89E+07	1.87E+07	<10^6
EF 4 V	9.42E+08	8.23E+08	7.39E+08	3.90E+08	6.91E+07	9.02E+07	<10^6
EF 20 pow	9.42E+08	6.93E+08	9.20E+07	3.87E+06	<10^6	<10^6	<10^6
EF 20 N2	9.42E+08	7.48E+08	8.21E+07	8.70E+06	<10^6	<10^6	<10^6
EF 20 V	9.42E+08	7.38E+08	8.32E+07	7.89E+06	<10^6	<10^6	<10^6
LM -20 pow	7.58E+08	8.90E+08	6.90E+08	5.09E+08	8.02E+07	1.89E+07	<10^6
LM -20 N2	7.58E+08	7.83E+08	7.02E+08	6.82E+08	2.01E+08	6.01E+07	8.78E+06
LM -20 V	7.58E+08	8.31E+08	7.89E+08	6.89E+08	1.82E+08	7.48E+07	6.70E+06
LM 4 pow	7.58E+08	7.39E+08	4.83E+08	7.92E+07	2.90E+07	<10^6	<10^6
LM 4 N2	7.58E+08	7.38E+08	6.72E+08	5.98E+08	8.91E+07	3.02E+07	<10^6
LM 4 V	7.58E+08	6.28E+08	6.98E+08	3.87E+08	7.83E+07	9.08E+06	<10^6
LM 20 pow	7.58E+08	3.29E+08	8.75E+07	6.48E+06	<10^6	<10^6	<10^6
LM 20 N2	7.58E+08	4.80E+08	9.83E+07	2.84E+07	<10^6	<10^6	<10^6
LM 20 V	7.58E+08	4.29E+08	8.31E+07	8.93E+06	<10^6	<10^6	<10^6
